# A qualitative analysis of follow-up interviews with SLE patients from the “living well with lupus” study

**DOI:** 10.3389/fmed.2025.1681780

**Published:** 2025-10-17

**Authors:** Fabiana Infante Smaira, Bruna Caruso Mazzolani, Sofia Mendes Sieczkowska, Marina Romero, Sandra Pasoto, Ana Lúcia de Sá Pinto, Fernanda Rodrigues Lima, Fabiana Braga Benatti, Hamilton Roschel, Mary Beth Weber, Bruno Gualano

**Affiliations:** ^1^Applied Physiology and Nutrition Research Group - School of Physical Education and Sport and Faculdade de Medicina FMUSP, Universidade de São Paulo, São Paulo, Brazil; ^2^Center of Lifestyle Medicine, Laboratory of Assessment and Conditioning in Rheumatology, Hospital das Clínicas HCFMUSP, Faculdade de Medicina FMUSP, Universidade de São Paulo, São Paulo, Brazil; ^3^School of Applied Sciences, Universidade Estadual de Campinas, São Paulo, Brazil; ^4^Hospital das Clinicas HCFMUSP, Faculdade de Medicina, Universidade de São Paulo, São Paulo, Brazil; ^5^Rheumatology Division, Faculdade de Medicina FMUSP, Universidade de São Paulo, São Paulo, Brazil; ^6^Hubert Department of Global Health (HDGH), Rollins School of Public Health, Emory University, Atlanta, Georgia

**Keywords:** follow-up, healthy eating, lifestyle intervention, physical activity, systemic lupus erythematosus

## Abstract

**Objectives:**

“Living Well with Lupus” (LWWL) consisted of a lifestyle intervention program tailored for patients with systemic lupus erythematosus (SLE) and high cardiovascular risk. In the present study, we assessed the maintenance of behavior changes related to physical activity and healthy eating after the 6-month LWWL program.

**Methods:**

Semi-structured interviews were conducted with 25 participants from the intervention group between 7 and 28 months after the program ended. Using qualitative content analysis, themes regarding behavior maintenance and perceived effects were identified.

**Results:**

Our findings suggest that maintaining the new lifestyle behaviors resulted in health benefits such as weight loss, pain reduction, and improved well-being; whereas worsening health, with increased anxiety, fatigue, and pain were reported among those that did not maintain the new behaviors over time. Most importantly, the main barriers to maintaining lifestyle changes included adverse weather conditions, family conflicts, health problems, and high work demands. On the other hand, family and professional support were highlighted as facilitators.

**Conclusion:**

These results suggest the importance of ongoing support to promote adherence to lifestyle changes in SLE patients. Integrated interventions with family and professional support are essential for sustaining these changes, highlighting the need for a holistic approach to health promotion for patients with chronic conditions.

## Introduction

Studies of individuals with systemic lupus erythematosus (SLE) reported improvements in inflammation, disease activity and symptoms, predisposition to infections, quality of life, functional capacity and some cardiometabolic risk factors (e.g., weight gain, high blood pressure, dyslipidemia) in response to isolated interventions aimed at improving either physical activity or eating behaviors (1–3). However, the efficacy and health effects of interventions combining both lifestyle behaviors were not investigated until the design and implementation of the “Living Well with Lupus” (LWWL) study (4).

While short-term improvements are encouraging, there is consistent evidence that lifestyle interventions often face challenges in sustaining long-term behavior change. In chronic conditions such as SLE, where inflammation and cardiovascular risk remain elevated throughout life, the persistence of healthy lifestyle behaviors is particularly relevant for disease management and prevention of comorbidities. Previous research in other chronic diseases has shown that initial gains from lifestyle programs may diminish over time without continued support, highlighting the need for follow-up assessments (5–7). Moreover, theoretical models of behavior change, such as the Transtheoretical Model, emphasize that relapse is common and that long-term maintenance is critical for genuine change (8). Therefore, understanding whether individuals are able to maintain new habits beyond the active intervention phase is critical to evaluating the true effectiveness and real-world applicability of self-care interventions.

The LWWL program was a 6-month behavioral intervention aimed at changing lifestyle behaviors, which includes a home-based exercise program and nutritional counseling. The intervention aimed to: (1) increase physical activity levels and reduce sedentary behavior; and (2) improve aspects of eating such as food consumption, eating structure, behaviors, and attitudes. The intervention group received the LWWL program in addition to standard care at the hospital’s SLE outpatient clinic. Standard care included the pharmacological management of SLE disease and its comorbidities, with general medical recommendations about a healthy lifestyle (e.g., “engage in more physical activities,” “restrict calorie intake,” “control your weight”). The detailed intervention protocol (4) as well as its effects on cardiovascular health (9) have been published elsewhere.

Qualitative analyses of the LWWL study showed that new behaviors and knowledge regarding physical activity and eating behaviors were achieved during the intervention period and that participants intended to maintain these behaviors (unpublished data). However, the Transtheoretical Model, widely cited in literature, posits that behavior change is only deemed to have genuinely occurred after a person has consistently practiced the new behaviors for 6 months (10). Based on this rationale, we conducted follow-up interviews with participants of the LWWL study to examine whether lifestyle behavior changes achieved during the intervention were maintained over time and to explore their perceived impact on health outcomes among SLE patients at high cardiovascular risk.

## Materials and methods

### Design and study sample

In this study, we used a descriptive qualitative research design employing semi-structured interviews to investigate the long-term effects of the intervention on lifestyle behaviors and health. Data consisted of individual interviews with SLE patients with high cardiovascular risk enrolled in the randomized controlled trial, (clinicaltrials.gov, NCT04431167) conducted at the Clinical Hospital (School of Medicine, University of Sao Paulo - Brazil) between August 2020 and March 2023. Participants were recruited from the “Living Well with Lupus” (LWWL) intervention group. This follow-up qualitative study is part of the broader “Living Well with Lupus” (LWWL) intervention program, which was originally designed as a randomized controlled trial.

The study was approved by the local Ethical Committee (Commission for Analysis of Research Projects, CAPPesq; approval: 19554719.5.0000.0068). Patients were required to sign an informed consent form before participating in the LWWL study.

### Recruitment and follow-up

Twenty-seven participants from the LWWL intervention group were invited to participate in the follow-up interview (one patient died in the period after the intervention study), conducted between 7 and 28 months after the intervention.

### Data collection

Initial explanations of the qualitative data collection design and purpose were done via text and audio messages and arrangements were individually made for preferred date/time for the in-depth interview.

Qualitative data was collected via semi-structured interviews conducted via WhatsApp and were audio-video recorded. Participants were reminded of recording procedures with assurance that transcription would be done in strict confidentiality and ensuring anonymity and were given the opportunity to ask questions prior to commencing the recorded interview. An interview guide was used, consisting of specific questions centered on evaluating patient’s lifestyle behaviors and health status (Supplementary material). The questions were created by three (BCM, FIS and SMS) researchers and were either approved or vetoed by two other researchers (BG and MBW), one with extensive expertise in lifestyle medicine and another in qualitative analysis, leading to the final questions used in the study. Only three questions, with accompanying probes, were used to avoid overburdening participants, resulting in relatively short interviews (5–15 min). Despite the short interview time, reviews of the data by the study team showed sufficient depth of the responses. Interviews were conducted by the three main researchers of the project (BCM, FIS and SMS).

Demographic and disease-related parameters were obtained through review of medical records and interviewing patients. Patients’ global health status and pain were assessed using the Visual Analogue Scale (VAS) in which patients graded their health status and pain using a 10-point scale. Height was measured with a stadiometer and weight with a calibrated scale after removing shoes. Intervention adherence was calculated as the percentage of study goals reached for reducing sedentary behavior (e.g., “stand up every hour at work”, “take a 20-min walk in the park on weekends”) and changing food consumption and other eating behaviors (i.e., structure, behavior and/or attitudes). Goal achievement was self-reported by participants. These data were collected during the 6-month LWWL trial.

### Data analysis

Quantitative data are presented as mean ± standard deviation for continuous variables or as frequency and percentage for categorical variables, unless otherwise stated. Given the qualitative nature of the study, no power calculation was performed. All feasible attempts to contact the eligible participants from the original trial were made, and no additional participants could be recruited beyond those who were interviewed, indicating that data saturation was achieved within the available sample. BCM, FIS and SMS transcribed audio recordings of the interviews verbatim and reviewed all transcriptions to ensure trustworthiness of the data. Analyses were conducted in Portuguese to ensure that linguistic nuance was maintained, and quotes were transcribed for inclusion in the manuscript. Deductive qualitative content analysis (11) was performed using the MAXQDA data management software to aid data coding and manipulation. An initial code system was developed based on the moderator’s guide and study objectives. After discussion with the study team, the code system was finalized (Supplementary material) and applied to the data by two research team members (BCM and FIS) iteratively; any differences in code usage were discussed and a consensus was reached by the coding team before finalizing the coding. A thematic analysis was conducted wherein thick descriptions of key themes around SLE patients’ current lifestyle behaviors and perceived health status were developed. Team discussions grouped the codes in the following themes: (a) multiple barriers and facilitators to lifestyle behaviors change maintenance; (b) maintenance of lifestyle behaviors change; and (c) perceived effects of maintaining lifestyle behaviors change.

## Results

### Participant characteristics

Of the 27 participants from the LWWL group invited to interview, 25 participated, 1 did not answer contact attempts, and 1 could not attend meetings at any of the proposed dates/times. Table 1 shows demographic and clinical characteristics of the participants. Patients’ average age and body mass index (BMI) were 41 ± 9 years and 28.0 ± 2.6, respectively. Most patients were from socioeconomic class D/E (the lowest socioeconomic class in Brazil), had completed high school or academic degrees and were employed. Disease activity and organ damage index were mild (Systemic Lupus Erythematosus Disease Activity Index [SLEDAI]: 0.7 ± 1.6). Also, moderate global health (VAS: 4.7 ± 2.8) and pain were reported (VAS: 4.4 ± 2.8). Disease-modifying anti-rheumatic drugs (DMARDs), immunosuppressants and corticoids were the drugs used in the pharmacological treatment for most patients.

**TABLE 1:** Demographic and clinical characteristics of the LWWL program participants.

Variables	Follow-up interview (*n* = 25)
	Mean ± SD or *n* (%)
Age (years)	41 ± 9
BMI (kg/m2)	28.0 ± 2.6
Working (Yes)	16 (64)
**Socioeconomic class**	
Class A	0 (0)
Class B	3 (12.5)
Class C	8 (33.3)
Class D/E	13 (54.2)
**Marital status**	
With a partner	16 (64)
Without a partner	9 (36)
**Nível educacional**	
Elementary school Complete/Incomplete	6 (24)
High school/Academic degree	19 (76)
**Disease parameters**	
Duration (years)	13.2 ± 7.8
SLEDAI	0.7 ± 1.6
SLICC/SDI AR	0.6 ± 1.3
VAS global health (0–10)	4.7 ± 2.8
VAS pain (0–10)	4.4 ± 2.8
**Pharmacological treatment and supplements**	
Biological drugs	5 (20)
DMARDs	17 (68)
NSAIDs	1 (4)
Glucocorticoids	15 (60)
Immunosuppressants	15 (60)
Muscle Relaxant	2 (8)
Painkillers	4 (16)
Antihypertensives	12 (48)
Statins	6 (24)
Anticoagulants	2 (8)
Antidiabetics	1 (4)
Antidepressants	9 (36)
Calcium	3 (12)
Vitamin D	16 (64)

Data expressed as unadjusted mean ± SD or *n* (%). BMI, body mass index; SLEDAI, Systemic Lupus Erythematosus Disease Activity Index; SLICC, Systemic Lupus International Collaborating Clinics/American College of Rheumatology – Damage Index; VAS, visual analogue scale; DMARDs, disease-modifying antirheumatic drugs; NSAIDs, non-steroidal anti-inflammatory drugs; LWWL, Living Well with Lupus.

Average intervention adherence was 71.5% (min.: 34.6; max.: 99.6%), with 74.3% of participants (min.: 29.2; max.: 100) reducing sedentary behavior, 65.0% (min.:18.1; max.: 100) doing the home-based exercise program and 74.5% (min.: 23.3; max.: 100) reporting dietary improvements. Twelve patients had high adherence (i.e., above 75% of goals reached), 12 had low adherence (i.e., below 75% of goals reached) and 1 was considered non-adherent (i.e., below 35% of goals reached).

### Maintenance of lifestyle behaviors changes

Twenty-three participants (92%) reported that they maintained some behaviors acquired during the intervention regardless of intervention adherence and time between the end of the study and the interview. Thirteen participants continued engaged with some regular physical exercise, 21 with healthier eating behaviors, and 13 with adding activity to break up sedentary time in their routines. Also, 17 participants reported maintaining multiple health behaviors, related to at least two of the following: exercising, reducing sedentary behaviors and dietary improvements.

Although participants report an understanding of the benefits of physical exercising and breaking sedentary behavior, some, but not all participants, maintained exercising long-term. Moreover, there was an increased focus not only on increasing physical activity but also on reducing time spent on sedentary behaviors (P1: “Now I sit, stand, and walk all the time. I don’t stay still anymore”). Strategies to avoid prolonged periods of sedentary behavior were developed among the patients (e.g., climbing stairs or taking short walks throughout the day), making the practice of unstructured physical activity realistic and easy to implement.

Regarding eating behaviors, participants reported a significant decrease in the consumption of ultraprocessed foods and added sugars, and maintenance of adequate water intake after the intervention. Furthermore, the participants demonstrated an understanding that while a healthy diet consists of eating less processed and more natural foods, accommodating exceptions (e.g., allowing for occasional treats) makes it easier to adopt these changes long-term.

In addition to changes in physical activity and eating related behaviors, which were the intervention focus, other lifestyle related behaviors (e.g., smoking cessation, starting therapy) were adapted during the intervention period and maintained afterward. Some patients reported starting these changes after the intervention was completed, because the intervention gave them the skills to make other health improvements.

### Multiple barriers and facilitators to lifestyle behaviors change maintenance

Participants described barriers and facilitators to maintenance of lifestyle behavior changes. Some participants were unable to maintain these behaviors due to the cold weather or sudden changes in temperature, which is commonly associated with worsening disease symptoms among patients with SLE.

*Ah, when it’s really cold, you know? For example, on a cold day like yesterday, like today, cloudy, I already feel a difference in my body, you know, I feel a change, but the colder it gets, the worse it gets. Then I don’t feel like doing anything, because I feel a lot of pain in my joints. Sometimes everything hurts (P17).*

Moreover, family and health problems (e.g., emergency surgery, caring for unwell parents) were also mentioned as important barriers to maintenance the lifestyle change. Health problems directly affect the motivation to start exercising while family barriers acted as a limiting factor in carrying out the exercises. Similarly, work-related issues (high demands at work, changes in work routine or duties/position, double shift load) caused excessive tiredness and a lack of time that compromised maintaining physical exercise goals. For many participants, these barriers worked in combination to inhibit behavior changes.

*Because I’m in a job right now that, you know, when you don’t have the right schedule, it’s still a bit of a mess, and we’re talking to resolve these issues, because the boss has moved, the child has changed schools, so I’m trying to figure out the right time for me to exercise now, because of these changes. [*…*] Some days I arrive late, some days I arrive early (P18).*

Despite being a barrier to implementing behavior changes, family encouragement was reported as a main motivating factor for maintaining the new behaviors. Professional support was also a primary motivating factor for maintain changes. For example, some participants exercised with family members while others worked with health professionals to maintain changes. One participant shared: “I thought it would be better to pay a private nutritionist who would follow me, I would send her messages, she would make video calls, so the follow-up was much better. [*not part of the original program, but a personal initiative inspired by the study*]. And that helped me a lot, you know?” (P22). Having someone who participates with or supports the participant in the process of behavior change contributes to less failure and consequently greater adherence.

### Perceived effects of maintaining lifestyle behaviors changes

Participants who maintained some or both physical activity and eating behaviors changes after the intervention reported good health and well-being in general, more willingness to do daily activities, less anxiety, weight loss/changes in body shape, and improvement of joint and back pain; for example, “I lost weight, but it doesn’t show on the scale, right? But when I put on some clothes, I saw that hydrotherapy [*not part of the original program, but a personal initiative inspired by the study*] really helps us lose weight.” (P1). Most participants credited the intervention with these changes, although a few participants attributed reduced anxiety and depression and increased motivation for daily activities to factors such as changing jobs and starting therapy. Among participants who had reported maintaining lifestyle changes but who also had worsening health symptoms, such as lupus flares or the discovery of new comorbidities, poor health outcomes were not felt to be related to continued adherence to lifestyle changes.

On the other hand, the majority of participants who were unable to maintain the behavior changes after the intervention reported a decline in health and well-being, including increased anxiety, fatigue, poorer sleep quality, weight gain, and heightened pain. Participants attributed these negative outcomes to their inability to adhere to the lifestyle changes introduced during the program.

## Discussion

Herein we described the long-term effects of the “Living Well with lupus” intervention on maintenance of lifestyle behaviors and its effects on health of SLE patients. The main findings were: (i) participants identified common barriers to maintaining lifestyle changes, such as adverse weather conditions, family conflicts, health problems, and high work demands, while citing family and professional support as key facilitators; (ii) Twenty-three participants successfully sustained changes in their eating habits, physical activity, or both; (iii) Overall, participants who maintained these behavior changes reported experiencing positive health-related outcomes.

Participants reported various barriers to maintain changes in their lifestyle behaviors. Among the difficulties, climatic factors such as adverse conditions and low temperatures were frequently mentioned. Patients with rheumatic disease face significant challenges to exercise during cold weather, as this can exacerbate joint pain and stiffness (12). Additionally, family conflicts and health problems (i.e., stressful events) also emerged as important barriers, a finding that aligns with the broader literature on chronic conditions, where such stressors are known to significantly impact family dynamics (13, 14). A meta-analysis of 122 studies aiming to correlate structural or functional social support with patient adherence to medical regimens, showed that family conflicts decreased adherence to treatment, with a risk of non-adherence 1.53 higher if there is high conflict in patient’s family than if there is not (15).

High work demands are another critical obstacle for changes in lifestyle behaviors, as the resulting stress and fatigue make it difficult to implement health changes (16). Evidence shows that fatigue and stress resulting from demanding work environments hinder individuals’ ability to prioritize health changes, as they often lack the necessary energy and motivation to engage in physical activity (16). Therefore, to foster healthier lifestyle choices, it is crucial to address the impact of occupational stress and its effects on decision-making processes related to exercise and well-being (16).

Conversely, participants’ reports show that family and professional support emerged as positive factors in maintaining lifestyle behaviors changes. Research shows that having family members who encourage healthy choices increases adherence to positive behavior changes (17). In patients with chronic illnesses, family routines and rituals play an even more crucial role in promoting emotional well-being and adherence to healthier health behaviors, since structuring family routines provides a sense of predictability and emotional support, while implementing rituals can strengthen family bonds, facilitating communication and cooperation around disease management (18). Similarly, support from health professionals is crucial, as they provide guidance and motivation, helping individuals overcome barriers, while the scarcity of such support may limit the effectiveness of interventions (19).

Most participants were able to maintain changes in their eating and physical activity behaviors, but some faced difficulties after the intervention ended. Participants who maintained most of the behavior changes reported positive health outcomes, which they attributed to the behavior changes made as part of the LWWL intervention. In contrast, participants who were unable to maintain the behavior changes reported negative health outcomes, which they attributed to their inability to sustain these behaviors. Our findings align with the existing literature, which demonstrates that engaging in and maintaining physical activity, (20, 21) reducing sedentary behavior, (22) and adhering to a healthier diet (23–25) improve physical and mental health, as well as overall quality of life. Additionally, in other studies, participants who maintained lifestyle behaviors also demonstrated a lower incidence of chronic conditions, such as type 2 diabetes, suggesting that maintaining healthy behaviors significantly contributes to disease prevention and the promotion of long-term optimal health (26).

Some participants who were less successful in reaching study goals or maintaining behavior changes long term, still reported health improvements at follow-up. However, these participants credited factors outside of the intervention activities, such as starting therapy or routine changes, for their improvements in health. Future interventions should consider using a more integrated, holistic approach, including both health behavior change education, mental health support, and resources for lifestyle improvement, to try and maximize outcomes for participants.

The strengths of this study lie in its qualitative approach, which effectively highlights the barriers and facilitators that participants face in maintaining their newly acquired behaviors. This provides valuable insights that can inform clinical practice regarding lifestyle changes and guide future research in this population. However, this study has some limitations: (1) The small sample size limits the generalizability of the findings; (2) the interviews were conducted by the same researchers who implemented the intervention, which may introduce bias; (3) as the follow-up study was planned after the intervention had concluded, interviews were conducted at varying intervals. This variation was attributable to participants’ availability, the impact of the pandemic and other logistical constraints, but also reflected the fact that participants had been continuously randomized into the main trial over a ∼2-year period, which naturally resulted in different lengths of time since completion of the intervention. The range of follow-up intervals allowed for the capture of participants’ experiences at different stages of behavior maintenance, but the heterogeneity in follow-up timing may have introduced recall bias, as participants’ accounts could differ according to the time since the intervention; and (4) the study relied on self-reported data obtained through semi-structured interviews, which are subject to recall bias, social desirability bias, and subjective interpretation. Nonetheless, this approach was consistent with the study’s objective of capturing participants’ own perceptions, meanings, and lived experiences after the intervention, and triangulation with other data sources was not considered appropriate, as it would have shifted the focus away from this subjective perspective.

The findings of this study suggests the importance of behavioral interventions in promoting sustainable lifestyle changes for patients with SLE and high cardiovascular risk. Most participants successfully maintained healthy habits following the intervention, resulting in improved overall health, reduced pain, and enhanced emotional well-being. However, challenges significantly hindered long-term adherence.

The LWWL intervention had a lasting positive impact on many participants’ health behaviors and well-being. However, sustained support addressing environmental, occupational, and psychosocial barriers is crucial to enhance long-term adherence. Future studies or programs may assist participants in overcoming these challenges and in leveraging family and professional support, which are key facilitators of long-term success, to achieve a more sustained impact. These findings underscore the need for continuous support strategies that integrate social and clinical components to ensure lasting positive changes. Future research can explore personalized and sustainable approaches aimed at optimizing long-term adherence and enhancing the quality of life for patients with SLE.

## Data Availability

The original contributions presented in this study are included in this article/Supplementary material, further inquiries can be directed to the corresponding author.
