# Supplementary material for: A qualitative analysis of follow-up interviews with SLE patients from the “living well with lupus” study
Source: Front Med (Lausanne). 2025 Oct 17;12:1681780. doi: 10.3389/fmed.2025.1681780 (PMC12575327; doi:10.3389/fmed.2025.1681780)
Supplement: Supplementary file 1 [file Data_Sheet_1.pdf]

SUPPLEMENTARY MATERIAL 1. Semi-Structured Interview Guide

1. What can you tell us about your life during the period between the end of the intervention and now?
2. Have you maintained the changes made during the intervention? If so, which ones and how?
3. How have you been feeling about your health recently? [physical health, mental health, quality of life, and well-being]

SUPPLEMENTARY MATERIAL 2. Focus Group Codebook for Thematic Analysis

Mnemonic or abbreviation	Maintenance of acquired physical activity and eating behaviors after intervention
Short description (code name)	Physical activity and eating behaviors maintenance
Detailed description	Reports about continuing or not with the behavioral changes (physical activity and eating) made during the intervention.
Inclusion criteria	Reports about maintaining behaviors (physical activity and eating) conquered during the intervention or not, how they did it, how confident they feel to continue doing it or start doing it again (for those who stopped), start other types of physical activity or nutritional monitoring.
Exclusion criteria	Maintenance of changes in other lifestyle behaviors.
Examples	*"Well, I started exercising, I signed up at a gym, since March, not March, I've been doing it since April."; "Well, I continue like this, avoiding meat. I go two or three days without eating meat."; "I try to do physical activity at least 3 times a week, regardless of what the practice is, I do some kind of activity. And I eat mainly processed foods, which I have been avoiding. I have actually been eating less. And sugar."*

Mnemonic or abbreviation	Maintenance or change in other lifestyle behaviors after intervention
Short description (code name)	Other lifestyle behaviors changes
Detailed description	Reports about continuing or not with the behavioral changes in other lifestyle behaviors (smoking, self-care) made during the intervention.

Inclusion criteria	Reports about maintaining behaviors conquered during the intervention or not, how they did it, how confident they feel to continue doing it or start doing it again (for those who stopped), start doing other positive changes in lifestyle behaviors.
Exclusion criteria	Maintenance of changes in physical activity and eating behaviors or behaviors not related to lifestyle.
Examples	*"I'm doing therapy..."; "After our, let's say, treatment that we had, I stopped smoking..."*

Mnemonic or abbreviation	Health related issues after intervention
Short description (code name)	Current health status
Detailed description	Reports about symptoms and complications of the disease and other comorbidities.
Inclusion criteria	Current status of physical health-related outcomes (pain, SLE-symptoms, cardiometabolic parameters, weight and body shape changes) and medication use.
Exclusion criteria	Current status of quality of life, mental health and routine.
Examples	*"I'm feeling a lot of pain in my back"; "I even gained 2kg."; "I'm just experiencing the beginning of osteoporosis in my spine."*

Mnemonic or abbreviation	Quality of life and well-being after intervention
Short description (code name)	Current quality of life and well-being status
Detailed description	How they felt and are feeling physically (fatigue, liveliness) and mentally (mood, stress, anxiety, and depression symptoms) after the intervention.
Inclusion criteria	The way participants feel about themselves physically and mentally after the intervention.
Exclusion criteria	Current health (physical and objective aspects) and routine status.
Examples	*"Willingness, I continue like this with will, I'm not lazy"; "I'm not doing very well mentally, you know?"; "I'm not sleeping well"*

Mnemonic or abbreviation	Routine and daily activities after intervention
Short description (code name)	Current routine and daily activities
Detailed description	How is their routine and how they are organizing and carrying out day-to-day activities after the intervention.
Inclusion criteria	Current routine, organization and ability to perform daily tasks.

Exclusion criteria	Current health (physical and objective aspects) and quality of life status.
Examples	*"What I used to be able to do, for example, climb a flight of stairs, nowadays if I climb them, I get very tired. I also get very tired when I catch the bus."; "Ah, although I'm a little more tired, but that's because of the routine, right? Like I said, I lived at my mom's house, I didn't have to do anything. So... A lot has changed, but..."*

Mnemonic or abbreviation	Facilitators for continuing lifestyle behaviors changes
Short description (code name)	Facilitators
Detailed description	Intervention or personal characteristics that facilitated continuing lifestyle behaviors changes
Inclusion criteria	Social, economic, environmental, physical, emotional, and intervention-related factors that facilitated continuing lifestyle behaviors changes.
Exclusion criteria	Factors that made it difficult to continue lifestyle behaviors changes.
Examples	*"Now we have changed our place of work, and now accessibility to food is much better."; "And now also with this bigger goal now that I want to get pregnant now, right?"*

Mnemonic or abbreviation	Barriers for continuing lifestyle behaviors changes
Short description (code name)	Barriers
Detailed description	Intervention or personal characteristics that made it difficult continuing lifestyle behaviors changes.
Inclusion criteria	Social, economic, environmental, physical, emotional and intervention-related factors that made continuing lifestyle behaviors changes difficult.
Exclusion criteria	Factors that facilitated continuing lifestyle behaviors changes.
Examples	*"But actually, I have a sore hip, so I had to stop walking."; "There are days when it's raining or very cold, then I don't go"; "Because I'm working two jobs, so now I don't have time anymore"*

SUPPLEMENTARY MATERIAL 3. **Emergent Themes**

Theme	Codes
Maintenance of lifestyle behaviors changes	Physical activity and eating behaviors maintenance<br>Other lifestyle behaviors changes
Multiple barriers and facilitators to lifestyle behaviors changes maintenance	Facilitators<br>Barriers
Perceived effects of maintaining lifestyle behaviors changes	Current routine and daily activities<br>Current quality of life and well-being status<br>Current health status
